# Structural data of highly luminescent lanthanide complexes constructed by bis-tridentate ligand and as sensor for Et_2_O

**DOI:** 10.1016/j.dib.2018.08.046

**Published:** 2018-09-05

**Authors:** Kai Zheng, Li-Wen Ding, Zi-Qi Liu, Ting Tang, Qing-Rong Yang

**Affiliations:** College of Chemistry and Chemical Engineering, Key Laboratory of Functional Small Organic Molecule, Ministry of Education and Jiangxi’s Key Laboratory of Green Chemistry, Jiangxi Normal University, Nanchang 330022, PR China

## Abstract

In this data article, we present the structural and PXRD data of the lanthanide complexes constructed by bis-tridentate ligand tppz (2,3,5,6-tetra-2-pyridinylpyrazine). Detailed structure, luminescence and sensing properties were discussed in “highly luminescent lanthanide complexes constructed by bis-tridentate ligand and as sensor for Et_2_O” (Zheng et al., 2018). The data includes the structure of Tb-complex, PXRD of Tb-complex, and also detailed structure information listed in Table 1, Table 2, Table 3.

**Specifications table**

**Table:** 

Subject area	*Chemistry*
More specific subject area	*Single crystal data of lanthanide complexes constructed by tppz*
Type of data	*Table, figure*
How data was acquired	*Crystallography open data base and crystallographic tool – Diamond : Crystallographic Information File Code: 1848709–1848711.cif*
Data format	*Analyzed*
Experimental factors	*Single crystal X-ray diffraction data was collected on a Bruker SMART 1000 CCD at 298(2) K, with Mo-Ka radiation (0.71073 Å) at room temperature. The structure was refined by full-matrix least-squares methods with SHELXL-97 module. The three single crystals are isostructural, they crystallize in triclinic space group P-1 (no. 2).*
Experimental features	*Block colorless single crystal.*
Data source location	*Jiangxi Normal University, Nanchang, China.*
Data accessibility	*The data are with this article.*
Related research article	K. Zheng, L.-W. Ding, C.-H. Zeng, highly luminescent lanthanide complexes constructed by bis-tridentate ligand and as sensor for Et_2_O, Inorg. Chem. Commun., 95 (2018) 95–99 [1].

**Value of the data**

• *This structure information would be valuable for further investigation of lanthanide complexes which constructed by tppz.*
• *This data would be valuable for the further investigation of the sensing properties.*
• *This data provide a new method to synthesize tridentate ligand coordinated lanthanide complexes.*

## 1 Data

**Fig. 1:**
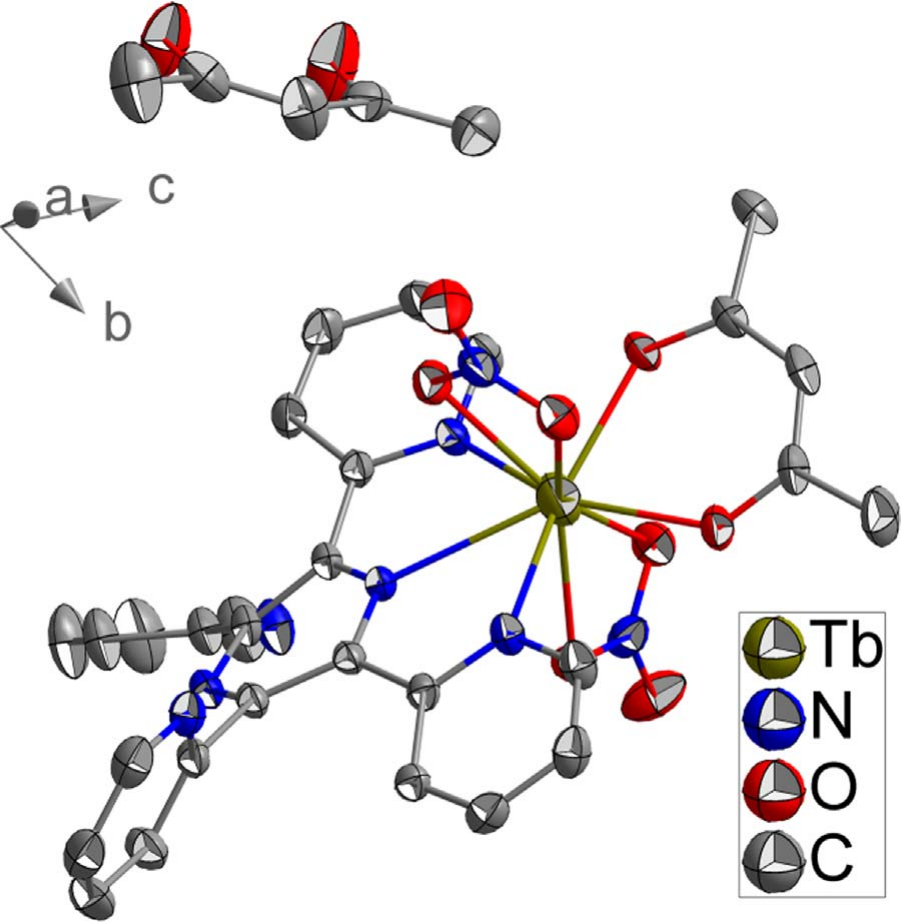
The structure shows the detailed structure information of **1a**.

**Fig. 2:**
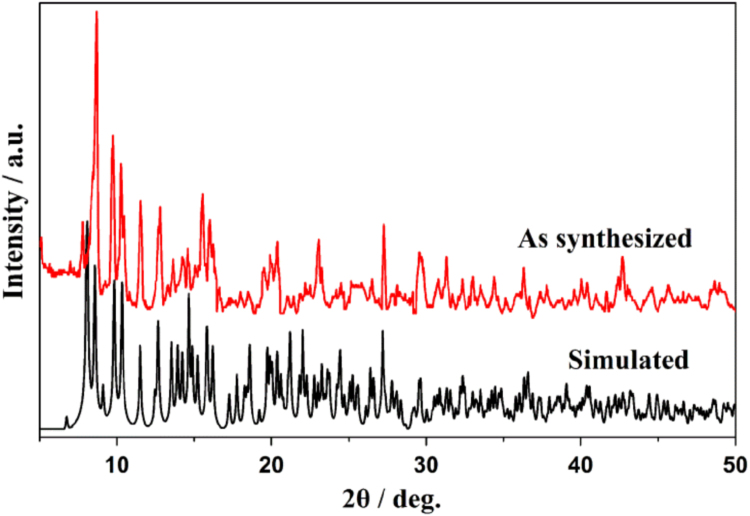
PXRD comparison of as synthesized **1a** and its simulated result.

The single crystal structures of isostructural **1a**–**1c** have the chemical formula of [Ln(tppz)(acac)(NO_3_)_2_]·acac (tppz = 2,3,5,6-tetra-2-pyridinylpyrazine; acac = acetylacetone; Ln^3+^ = Tb^3+^, **1a**; Er^3+^, **1b**; Y^3+^, **1c**). Since 1a–1c are isostructural, as an example, the crystal structure of **1a** is discussed in somewhat greater detail. As shown in Fig. 1, each unit contains one Tb^3+^, one tppz, two ${NO}_{3}^{-}$, one coordinated acac and one crystalline acac, to form an electroneutral unit. PXRD peak positions of bulk sample **1a** compete well with its simulated result, suggesting high phase purity of the as synthesized **1a** (Fig. 2) [2], [3], [4], [5], [6], [7], [8]. Bond lengths and angles for **1a–1c** are in line with the reported lanthanide complexes [9], [10], [11], [12], [13], [14], which are listed in Table 1, Table 2, Table 3.

**Table 1:** Bond lengths [Å] and bond angles [deg] for **1a**.

Tb(1)-O(7)	2.2733(18)	Tb(1)-O(2)	2.496(2)
Tb(1)-O(8)	2.279(2)	Tb(1)-N(1)	2.528(2)
Tb(1)-O(1)	2.438(2)	Tb(1)-N(3)	2.540(2)
Tb(1)-O(4)	2.454(2)	Tb(1)-N(2)	2.613(2)
Tb(1)-O(5)	2.484(2)		
O(7)-Tb(1)-O(8)	76.27(7)	O(7)-Tb(1)-N(2)	142.67(7)
O(7)-Tb(1)-O(1)	86.49(8)	O(8)-Tb(1)-N(2)	141.06(7)
O(8)-Tb(1)-O(1)	129.60(8)	O(1)-Tb(1)-N(2)	68.90(7)
O(7)-Tb(1)-O(4)	126.27(7)	O(4)-Tb(1)-N(2)	72.66(7)
O(8)-Tb(1)-O(4)	82.36(8)	O(5)-Tb(1)-N(2)	106.51(7)
O(1)-Tb(1)-O(4)	141.56(7)	O(2)-Tb(1)-N(2)	104.64(7)
O(7)-Tb(1)-O(5)	75.73(7)	N(1)-Tb(1)-N(2)	63.64(7)
O(8)-Tb(1)-O(5)	78.68(8)	N(3)-Tb(1)-N(2)	62.56(7)
O(1)-Tb(1)-O(5)	142.04(7)	O(7)-Tb(1)-N(8)	100.76(7)
O(4)-Tb(1)-O(5)	51.79(7)	O(8)-Tb(1)-N(8)	78.37(8)
O(7)-Tb(1)-O(2)	78.27(7)	O(1)-Tb(1)-N(8)	151.97(7)
O(8)-Tb(1)-O(2)	78.58(8)	O(4)-Tb(1)-N(8)	25.99(7)
O(1)-Tb(1)-O(2)	51.43(7)	O(5)-Tb(1)-N(8)	25.84(7)
O(4)-Tb(1)-O(2)	143.85(7)	O(2)-Tb(1)-N(8)	156.46(7)
O(5)-Tb(1)-O(2)	148.76(7)	N(1)-Tb(1)-N(8)	98.32(7)
O(7)-Tb(1)-N(1)	146.58(7)	N(3)-Tb(1)-N(8)	81.75(8)
O(8)-Tb(1)-N(1)	81.07(7)	N(2)-Tb(1)-N(8)	90.42(7)
O(1)-Tb(1)-N(1)	89.40(7)	O(7)-Tb(1)-N(7)	82.00(7)
O(4)-Tb(1)-N(1)	73.53(7)	O(8)-Tb(1)-N(7)	104.18(8)
O(5)-Tb(1)-N(1)	123.41(7)	O(1)-Tb(1)-N(7)	25.71(6)
O(2)-Tb(1)-N(1)	73.36(7)	O(4)-Tb(1)-N(7)	151.54(6)
O(7)-Tb(1)-N(3)	83.76(7)	O(5)-Tb(1)-N(7)	156.23(7)
O(8)-Tb(1)-N(3)	148.47(7)	O(2)-Tb(1)-N(7)	25.73(7)
O(1)-Tb(1)-N(3)	72.08(7)	N(1)-Tb(1)-N(7)	80.11(7)
O(4)-Tb(1)-N(3)	90.35(8)	N(3)-Tb(1)-N(7)	96.79(8)
O(5)-Tb(1)-N(3)	72.84(8)	N(2)-Tb(1)-N(7)	86.27(7)
O(2)-Tb(1)-N(3)	121.14(7)	N(8)-Tb(1)-N(7)	176.69(6)
N(1)-Tb(1)-N(3)	126.20(7)

**Table 2:** Bond lengths [Å] and bond angles [deg] for **1b**.

Er(2)-O(7)	2.2462(19)	Er(2)-O(2)	2.463(2)
Er(2)-O(8)	2.249(2)	Er(2)-N(3)	2.496(2)
Er(2)-O(1)	2.400(2)	Er(2)-N(1)	2.505(2)
Er(2)-O(5)	2.414(2)	Er(2)-N(2)	2.566(2)
Er(2)-O(4)	2.457(2)		
O(7)-Er(2)-O(8)	77.53(8)	O(7)-Er(2)-N(2)	141.84(7)
O(7)-Er(2)-O(1)	84.35(8)	O(8)-Er(2)-N(2)	140.64(7)
O(8)-Er(2)-O(1)	129.69(8)	O(1)-Er(2)-N(2)	70.00(8)
O(7)-Er(2)-O(5)	127.35(7)	O(5)-Er(2)-N(2)	72.79(7)
O(8)-Er(2)-O(5)	81.23(8)	O(4)-Er(2)-N(2)	107.03(7)
O(1)-Er(2)-O(5)	142.80(7)	O(2)-Er(2)-N(2)	105.70(8)
O(7)-Er(2)-O(4)	75.86(8)	N(3)-Er(2)-N(2)	64.33(7)
O(8)-Er(2)-O(4)	77.96(9)	N(1)-Er(2)-N(2)	63.45(7)
O(1)-Er(2)-O(4)	141.49(7)	O(7)-Er(2)-N(8)	101.28(8)
O(5)-Er(2)-O(4)	52.70(7)	O(8)-Er(2)-N(8)	77.04(8)
O(7)-Er(2)-O(2)	77.49(8)	O(1)-Er(2)-N(8)	153.09(7)
O(8)-Er(2)-O(2)	77.95(8)	O(5)-Er(2)-N(8)	26.49(7)
O(1)-Er(2)-O(2)	52.28(7)	O(4)-Er(2)-N(8)	26.27(7)
O(5)-Er(2)-O(2)	142.67(7)	O(2)-Er(2)-N(8)	154.59(7)
O(4)-Er(2)-O(2)	147.22(8)	N(3)-Er(2)-N(8)	98.82(8)
O(7)-Er(2)-N(3)	145.81(7)	N(1)-Er(2)-N(8)	82.06(8)
O(8)-Er(2)-N(3)	80.43(8)	N(2)-Er(2)-N(8)	90.98(7)
O(1)-Er(2)-N(3)	90.05(8)	O(7)-Er(2)-N(7)	79.90(8)
O(5)-Er(2)-N(3)	73.68(7)	O(8)-Er(2)-N(7)	103.80(9)
O(4)-Er(2)-N(3)	124.31(7)	O(1)-Er(2)-N(7)	26.19(7)
O(2)-Er(2)-N(3)	72.55(8)	O(5)-Er(2)-N(7)	152.42(7)
O(7)-Er(2)-N(1)	82.45(7)	O(4)-Er(2)-N(7)	154.73(7)
O(8)-Er(2)-N(1)	147.42(8)	O(2)-Er(2)-N(7)	26.09(7)
O(1)-Er(2)-N(1)	72.54(8)	N(3)-Er(2)-N(7)	80.38(7)
O(5)-Er(2)-N(1)	91.08(8)	N(1)-Er(2)-N(7)	97.59(8)
O(4)-Er(2)-N(1)	72.35(8)	N(2)-Er(2)-N(7)	87.75(8)
O(2)-Er(2)-N(1)	122.38(8)	N(8)-Er(2)-N(7)	178.70(6)
N(3)-Er(2)-N(1)	127.78(7)

**Table 3:** Bond lengths [Å] and bond angles [deg] for **1c**.

Y(2)-O(7)	2.2537(19)	Y(2)-O(4)	2.473(2)
Y(2)-O(8)	2.259(2)	Y(2)-N(3)	2.510(2)
Y(2)-O(5)	2.409(2)	Y(2)-N(1)	2.527(2)
Y(2)-O(2)	2.421(2)	Y(2)-N(2)	2.587(2)
Y(2)-O(1)	2.459(2)		
O(7)-Y(2)-O(8)	77.10(7)	O(5)-Y(2)-N(1)	72.14(8)
O(7)-Y(2)-O(5)	85.12(8)	O(2)-Y(2)-N(1)	90.94(8)
O(8)-Y(2)-O(5)	129.74(8)	O(1)-Y(2)-N(1)	72.43(8)
O(7)-Y(2)-O(2)	126.91(7)	O(4)-Y(2)-N(1)	121.94(7)
O(8)-Y(2)-O(2)	81.59(8)	N(3)-Y(2)-N(1)	127.08(7)
O(5)-Y(2)-O(2)	142.36(7)	O(7)-Y(2)-N(2)	142.02(7)
O(7)-Y(2)-O(1)	75.77(8)	O(8)-Y(2)-N(2)	140.88(7)
O(8)-Y(2)-O(1)	78.46(8)	O(5)-Y(2)-N(2)	69.47(7)
O(5)-Y(2)-O(1)	141.39(7)	O(2)-Y(2)-N(2)	72.89(7)
O(2)-Y(2)-O(1)	52.42(7)	O(1)-Y(2)-N(2)	106.74(7)
O(7)-Y(2)-O(4)	78.18(7)	O(4)-Y(2)-N(2)	104.99(7)
O(8)-Y(2)-O(4)	78.23(8)	N(3)-Y(2)-N(2)	64.09(7)
O(5)-Y(2)-O(4)	52.06(7)	N(1)-Y(2)-N(2)	62.99(7)
O(2)-Y(2)-O(4)	142.80(7)	O(7)-Y(2)-N(5)	100.98(8)
O(1)-Y(2)-O(4)	148.21(7)	O(8)-Y(2)-N(5)	77.59(8)
O(7)-Y(2)-N(3)	146.21(7)	O(5)-Y(2)-N(5)	152.52(7)
O(8)-Y(2)-N(3)	80.69(7)	O(2)-Y(2)-N(5)	26.39(7)
O(5)-Y(2)-N(3)	89.91(7)	O(1)-Y(2)-N(5)	26.09(7)
O(2)-Y(2)-N(3)	73.56(7)	O(4)-Y(2)-N(5)	155.34(7)
O(1)-Y(2)-N(3)	124.07(7)	N(3)-Y(2)-N(5)	98.70(7)
O(4)-Y(2)-N(3)	72.56(7)	N(1)-Y(2)-N(5)	81.97(8)
O(7)-Y(2)-N(1)	82.93(7)	N(2)-Y(2)-N(5)	90.86(7)
O(8)-Y(2)-N(1)	147.94(7)		

## 2 Experimental design, materials, and methods

The three lanthanide complexes **1a–1c** were synthesized with similar procedures, the molar ratio of tppz : Ln(NO_3_)_3_·6H_2_O ≈ 3 : 2, 0.327 mmol tppz was dissolved in 40 mL CHCl_3_ and Ln(NO_3_)_3_·6H_2_O (0.214 mmol) dissolved in 20 mL acac, the two solutions were mixed together and let stand for 12 h, the mixture was filtered and the filtrate evaporated in a quiet environment. Four weeks later, crystals suitable for single crystal X-ray test were obtained by filtration [1].

Single crystal X-ray diffraction data was tested on a Bruker SMART 1000 CCD, with Mo-Ka radiation (Wavelength = 0.71073 Å) at room temperature. The structure was refined by full-matrix least-squares methods with SHELXL-97 module. Phase purity of bulk sample was determined on a DMAX2200VPC diffractometer [2].
