# Supplementary material for: Structural data of highly luminescent lanthanide complexes constructed by bis-tridentate ligand and as sensor for Et2O
Source: Data Brief. 2018 Sep 5;20:1428–33. doi: 10.1016/j.dib.2018.08.046 (PMC6148709; doi:10.1016/j.dib.2018.08.046)
Supplement: Supplementary file 1 — Supplementary material [file mmc1.docx]

Dear Editor,

I am here representing all the authors to declare that this manuscript (DIB-D-18-01809R1) has no competing financial interest or other conflit of interest.

Mrs Li-Wen Ding

------------------------------
